# ﻿New species and other new records of the family Mycetophilidae (Insecta, Diptera) from Morocco

**DOI:** 10.3897/zookeys.1197.118503

**Published:** 2024-04-17

**Authors:** Mohamed Amin El Mouden, Peter J. Chandler, Ouafaa Driauach, Ouarda Banamar, Imane Saidoun, Abdellatif Akarid, Khalid Aattouch, Boutaïna Belqat

**Affiliations:** 1 LESCB URL/CNRST N°18, Faculty of Sciences, Abdelmalek Essaâdi University, Tétouan, Morocco Abdelmalek Essaâdi University Tétouan Morocco; 2 606B Berryfield Lane, Melksham, Wilts SN12 6EL, UK Unaffiliated Wilts United Kingdom; 3 Biotechnology, Environmental Technology and Valorization of Bio-Resources Team, Department of Biology, Laboratory of Research and Development in Engineering Sciences Faculty of Sciences and Techniques Al-Hoceima, Abdelmalek Essaadi University, Tétouan, Morocco Abdelmalek Essaadi University Tétouan Morocco

**Keywords:** Fungus gnats, Moroccan endemism, North Africa

## Abstract

Twelve species in nine genera of Mycetophilidae are newly recorded from Morocco and from North Africa. Five species are described as new to science: *Rymosiaebejeri***sp. nov.**, *Leiaarcana***sp. nov.**, *Megophthalmidiaamsemlili***sp. nov.**, *Mycomyamira***sp. nov.**, and *Phthiniasnibbypinsae***sp. nov**. Three species are newly recorded from Gibraltar.

## ﻿Introduction

Banamar et al. (2020) included Moroccan records of 64 species of Mycetophilidae, of which 54 were newly recorded, but they noted that most of the species recorded are widespread in the Mediterranean region and more widely in Europe and the Palaearctic Region. However, also found were some species that were new to science. These and some other species identified after that publication are treated here. Results are presented in the same taxonomic order as by Banamar et al. (2020) and Kettani et al. (2022) in the Catalogue of Moroccan Diptera, except that *Docosia* Winnertz, 1864 is placed in subfamily Gnoristinae rather than Leiinae following recent phylogenetic studies using molecular methods (e.g. Kaspřák et al. 2019).

## ﻿Materials and methods

**Figure 1.**
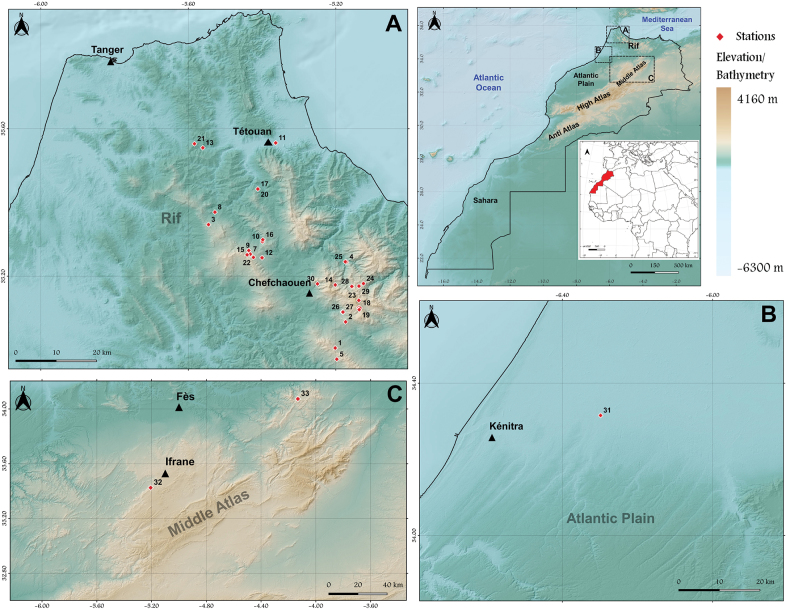
Maps showing all sampling localities for Mycetophilidae in the current study; numbers correspond to those in Table 1.

Most of the material totalling 148 specimens (122 males and 26 females) was collected using diverse techniques such as sweeping and rearing, by B. Belqat, O. Driauach, and M.A. El Mouden between 12 December 2013 and 28 February 2022 in 31 of 33 sites in the Rif and Middle Atlas Mountains, and on the Atlantic plain (Fig. 1; Table 1). Additional materials were collected in the Middle Atlas (8 May 2012) and the Rif (12 June 2013) were provided by Dr Martin Ebejer, who kindly permitted us to publish his new records.

**Table 1.** Sampling sites (in alphabetical order) hosting the species collected in the Rif and Middle Atlas Mountains and on the Atlantic plain, with localities, altitudes, and geographic coordinates. PNTLS = National Park of Talassemtane; PPNB = Bouhachem Natural Park Project; PNTZK = National Park of Tazekka.

Site	Locality	Elevation (m)	Latitude
Rif			
1. Aïn El Ma Bared	Bouzthate, Parc Bab El Karne	1267	35°00.333'N, 5°12.105'W
2. Aïn El Maounzil	PNTLS	1106	35°04.577'N, 5°10.406'W
3. Aïn Sidi Brahim Ben Arrif	PPNB	897	35°20.398'N, 5°32.712'W
4. Akchour	PNTLS	600	35°14.203'N, 5°10.145'W
5. Bab El Karne	Douar Tamakoute, Parc Bab El Karne	1248	34°58.510'N, 5°11.838'W
6. Bab Rouida	PNTLS	1678	35°06.881'N, 5°08.270'W
7. Daya Amsemlil	Jbel Bouhachem, PPNB	1059	35°15.596'N, 5°25.917'W
8. Daya avant Taïda	Taïda, PPNB	436	35°22.426'N, 5°31.662'W
9. Daya Mtahen	Jbel Bouhachem, PPNB	966	35°16.195'N, 5°26.158'W
10. Douar Bni Leit	Bni Leit, PPNB	836	35°17.382'N, 5°23.558'W
11. Faculté des Sciences	Université Abdelmalek Essaâdi, Tétouan	7	35°33.413'N, 5°21.464'W
12. Forêt Adayourha	PPNB	794	35°14.599'N, 5°24.001'W
13. Forêt Aïn Lahcen	Aïn Lahcen	186	35°32.532'N, 5°33.378'W
14. Forêt Azilane	Azilane, PNTLS	1291	35°10.354'N, 5°12.053'W
15. Forêt Bab Hajara	PPNB	1203	35°15.292'N, 5°26.258'W
16. Forêt Bni Leit	Bni Leit, PPNB	826	35°17.564'N, 5°23.527'W
17. Forêt Bouhachem st.1	PPNB	1016	35°16.119'N, 5°26.144'W
18. Forêt Jbel Lekrâa	PNTLS	1541	35°06.825'N, 5°08.077'W
19. Forêt Malâab Tizimezzan	PNTLS	1452	35°06.562'N, 5°08.197'W
20. Forêt Sed Nakhla	Barrage Nakhla, PPNB	414	35°26.110'N, 5°24.407'W
21. Khandek Melouka	Aïn Lahcen	287	35°33.326'N, 5°34.597'W
22. Maison forestière Bouhachem	PPNB	1048	35°15.040'N, 5°25.240'W
23. Maison forestière Talassemtane	PNTLS	1674	35°08.076'N, 5°08.262'W
24. Marabout Abou Bnar	Abou Bnar, PNTLS	1247	35°10.812'N, 5°07.500'W
25. Oued Ferda	Akoumi, PNTLS	420	35°14.350'N, 5°10.46'W
26. Oued Majjou	Majjou Village, PNTLS	799	35°06.186'N, 5°10.935'W
27. Oued Majjou avant source	Majjou Village, PNTLS	1055	35°06.105'N, 5°10.502'W
28. Pont Imezzane	Imezzane, PNTLS	1181	35°10.391'N, 5°09.353'W
29. Route vers Abou Bnar	Abou Bnar, PNTLS	1410	35°10.398'N, 5°08.234'W
30. Tissemlal	PNTLS	1187	35°10.458'N, 5°14.587'W
Atlantic plain			
31. Sidi Yahya El Gharb	Sidi Yahya El Gharb	25	34°18.552'N, 6°17.532'W
Middle Atlas			
32. Forêt-3.5 km S. Azrou	Azrou	1450	33°25.491'N, 5°12.393'W
33. Oued Taourirt	PNTZK	1343	34°04.225'N, 4°07.508'W

The holotypes of the newly described species and M.J. Ebejer collection will be deposited at the Natural History Museum, London, UK (**NHMUK**). Paratypes and additional studied materials will be deposited in our Diptera collection, in the Department of Biology, Faculty of Sciences of Tétouan, Abdelmalek Essaâdi University, Morocco (**UAE-FST**).

## ﻿Taxonomy

### ﻿Subfamily Mycetophilinae Newman, 1834

**Tribe Exechiini Edwards, 1925**

**Genus *Exechia* Winnertz, 1864**

#### ﻿ Exechia repandoides

Taxon classificationAnimaliaDipteraMycetophilidae

Caspers, 1984

6AA5A8A2-14BF-596B-BD9C-022DCF256A9F

##### New record.

Morocco – **Rif Region** • 2♂♂, 6♀♀; Faculté des Sciences; 14–28/II/2022; A. Akarid leg; reared from fungus *Cyclocybeaegerita*; UAE-FST R22/2441.

##### Comments.

**Figures 2, 3.**
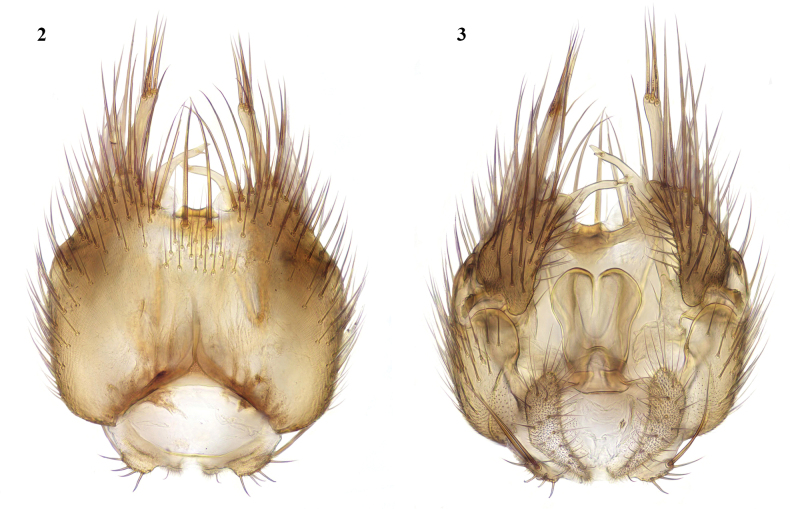
*Exechiarepandoides* Caspers, male terminalia **2** ventral view **3** dorsal view.

**Figure 4.**
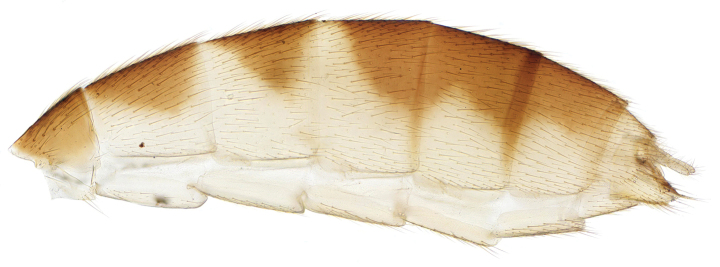
*Exechiarepandoides* Caspers, lateral view of female abdomen.

This species belongs to the *Exechiaparva* Lundström, 1909 group, which was revised by Lindemann et al. (2021). The Moroccan males are similar in structure of their terminalia (Figs 2, 3) to *E.repandoides*, which is widespread in central and northern Europe and is also recorded from Corsica. These Moroccan males differ in coloration from European specimens, which have the abdomen dark coloured apart from the yellow terminalia. The Moroccan males have the abdomen brown dorsally, but broadly yellow laterally on tergites 2 and 3; sternites 1–3 are yellow, while tergites and sternites 4–6 are brown. The female has not been previously associated for *E.repandoides*. The Moroccan females are more brightly coloured than European females of this group; the abdomen (Fig. 4) is broadly yellow laterally, with brown dorsal markings on tergites 2–6 often not quite reaching fore margins, while tergite 7, the ovipositor, and all sternites are yellow. New to North Africa.

**Genus *Exechiopsis* Tuomikoski, 1966**

#### ﻿ Exechiopsis corona

Taxon classificationAnimaliaDipteraMycetophilidae

Chandler & Ribeiro, 1995

131A22F7-9C9E-59E2-9DD9-1FEFB7E4F75E

##### New record.

Morocco – **Rif Region** • 1♂; Maison forestière, Talassemtane; 13/II/2020; B. Belqat and O. Driauach leg; collected using sweep net; UAE-FST R20/2442.

##### Comments.

This species was described from two males, respectively from Tenerife, Canary Islands and the Greek island of Naxos (Chandler and Ribeiro 1995). It has otherwise only been recorded from Cyprus (Chandler et al. 2006). New to North Africa.

**Genus *Rymosia* Winnertz, 1864**

#### ﻿ Rymosia ebejeri

Taxon classificationAnimaliaDipteraMycetophilidae

Chandler & Belqat sp. nov.

9123854A-2142-5A38-8A77-6E7C7E7A3854

https://zoobank.org/7E574E63-8756-4B7B-AB24-F62930656AF1

##### Type material.

***Holotype.*** Morocco – **Rif Region** • ♂ (mounted in DMHF); Forêt Jbel Lekraa; 12/VI/2013; M.J. Ebejer leg; collected using sweep net; NHMUK. **Paratypes.** Morocco – **Rif Region** • 1♂; Oued Majjou; 5/II/2019; B. Belqat and O. Driauach leg; collected using sweep net; UAE-FST R19/2425 • 3♂♂; Tissemlal; 3/II/2020; B. Belqat and O. Driauach leg; collected using sweep net; UAE-FST R20/2426 • 1♂; Bab Rouida; 13/II/2020; B. Belqat and O. Driauach leg; collected using sweep net; UAE-FST R202427.

The species has been found mostly in the PNTLS, at high altitudes, in the large expanse of a cedar forest, in the environment of the Majjou River and in/and around the depression in a rock, resembling a small cave in the Bab Rouida site.

##### Diagnosis.

This belongs among those *Rymosia* species without any spinules on the male fore tarsi. It is very distinct from other species in the structure of its male terminalia. The produced apical margin of the gonocoxites, bearing strong apical setae, is an especially unusual feature. This and the gonostylus concealed within the gonocoxites in ventral view distinguish it from the other three *Rymosia* species recorded from Morocco, *R.affinis* Winnertz, 1864, *R.beaucournui* Matile, 1963, and *R.pseudocretensis* Burghele-Balacesco, 1966 (Banamar et al. 2020).

##### Description.

**Figures 5–7.**
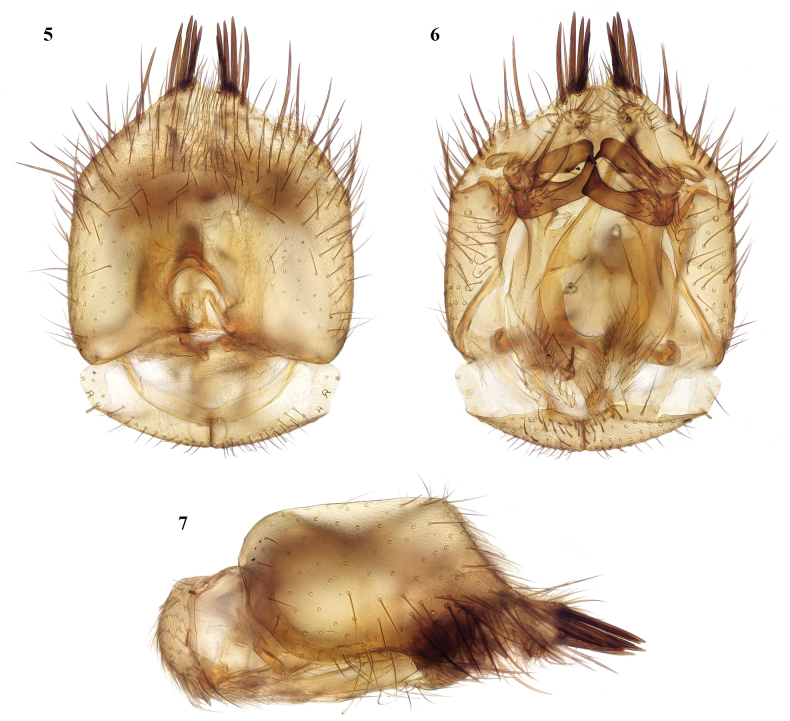
*Rymosiaebejeri* sp. nov., male terminalia **5** ventral view **6** dorsal view **7** lateral view.

**Male.** Wing length 4–4,5 mm. ***Coloration*.** Head brown, with face yellowish. Antenna with basal segments and base of first flagellomere yellow, flagellum otherwise brownish. Palpus yellow. Thorax yellowish brown; mesonotum with three more or less fused

brown stripes, leaving humeral area and sides yellowish. Legs all yellow. Wing clear yellowish. Abdomen yellow with apical half of tergites 2–5 brown, each of these markings extended forwards as a dorsal triangle almost reaching fore margin and as a rounded extension laterally on each side; tergite 6 all brown. Terminalia yellow. ***Head*.** Antenna longer than head and thorax together, with flagellomeres progressively longer, from 3–4× to 6× as long as broad. Palpus elongate. ***Thorax*.** Mesonotum with long, dark setae in dorsocentral rows, near the side margins, and on pronotum; one long dark proepisternal seta; anepisternum covered with short setae; laterotergite with several long setae medially. ***Legs*.** Without any modification of tarsi (found in some species of this genus); hind tibial spurs about a third as long as hind tarsomere 1; tibial setae short, not longer than width of tibia. ***Wing*.** Vein Sc short, ending free. Crossvein r-m 2–3× as long as stem of median fork. Base of posterior fork at or before level of base of median stem; false vein extends to level of about half length of posterior fork, vein CuP reaches to level of about a third of fork. ***Terminalia*** (Figs 5–7). Gonocoxites produced medially with a group of 4 or 5 short but strong spines on each side of a small median emargination, and a row of strong setae on the apical margin external to the spines; gonostylus with ventral lobe short, not extending beyond margin of gonocoxites, rounded and densely setose apically; median and dorsal lobes with long setae basally and an apically bare sclerotised portion adjacent to ventral lobe, dorsal lobe with long basal extension bare except for preapical spine.

**Female.** Unknown.

##### Etymology.

Named for Dr Martin Ebejer, who collected the first known specimen.

### ﻿Subfamily Leiinae Edwards, 1925

**Genus *Leia* Meigen, 1818**

With the species added here, four *Leia* species are known to occur in Morocco. Two of them, *L.beckeri* Landrock, 1940 and *L.arsona* Hutson, 1978 have a mainly Mediterranean distribution, and both have a dark marking over the r-m crossvein, and one behind the posterior fork, in addition to a preapical wing band; *L.arsona* differs from other species in having a dark knob to the halteres. *Leiabimaculata* (Meigen, 1804) is widespread in the Palaearctic Region; it has a preapical wing band but lacks any central marking. It is very variable in body coloration, from a largely black thorax and abdomen to being largely pale, but with bands on the abdominal tergites that are usually broader in the middle than at the sides. Moroccan specimens of *L.bimaculata* are generally lighter coloured, and it became apparent that some darker coloured Moroccan specimens also had differences in the male terminalia from typical *bimaculata*; we conclude here that these represent a distinct species that may have been overlooked elsewhere within the range of *L.bimaculata*. Polevoi and Salmela (2016) described and figured some variation in European specimens of *L.bimaculata*, with some specimens from Finland and Russia differing in details of the male terminalia including lack of a dorsal projection at the base of the gonostylus; they showed similar variation in body coloration to typical *L.bimaculata* but had unmarked wings. Further study of *L.bimaculata* across its range is necessary to establish whether more species may have been overlooked under this name.

#### ﻿ Leia arcana

Taxon classificationAnimaliaDipteraMycetophilidae

Chandler, Belqat & Driauach sp. nov.

03062551-BE71-5AFF-87FC-932720F2F9A0

https://zoobank.org/46D501E8-0AEA-4D5E-841A-0A40BC967CFB

##### Type material.

***Holotype*.** Morocco – **Rif Region** • ♂ (mounted in DMHF from alcohol, terminalia on a slide); Aïn El Maounzil; 3/II/2020; B. Belqat and O. Driauach leg; collected using sweep net; NHMUK. ***Paratypes.*** Morocco – **Rif Region** • 3♂♂; Aïn Sidi Brahim Ben Arrif; 25/IV/2014. B. Belqat and O. Driauach leg; collected using sweep net; UAE-FST R14/2401 • 1♂; Maison forestière, Talassemtane; 17/VI/2014; B. Belqat and O. Driauach leg; collected using sweep net; UAE-FST R142402 • 1♂; Aïn El Ma Bared; 25/XII/2015; B. Belqat and O. Driauach leg; collected using sweep net; UAE-FST R15/2403 • 1♂; Bab El Karne; 25/XII/2015; B. Belqat and O. Driauach leg; collected using sweep net; UAE-FST R15/2404 • 1♂; Daya Mtahen; 23/III/2021; B. Belqat and O. Driauach leg; collected using sweep net; UAE-FST R21/2405.

##### Other material.

Morocco – **Rif Region** • 1♀; Aïn Sidi Brahim Ben Arrif; 25/IV/2014; B. Belqat and O. Driauach leg; collected using sweep net; UAE-FST R14/2406. – **Middle Atlas Region** • 1♀; Forêt–3.5 km S. Azrou; cedar forest; 8/V/2012; M.J. Ebejer leg; collected using sweep net; NHMUK.

This species was collected mostly in environments of aquatic ecosystems such as springs (Aïn) and ponds (Daya), but also in forest environments.

##### Diagnosis.

The most obvious differences in the male terminalia from *L.bimaculata* (Figs 10, 13, 16) are that the apical part of the gonostylus is shorter and thicker, and the adjacent ventral lobe of the gonocoxites is broader basally (arrowed in Fig. 15 of *L.arcana* and in Fig. 16 of *L.bimaculata* for comparison). In these respects, *L.arcana* resembles these structures in the figures of *L.montanosilvatica* Zaitzev, 1994, described from Kyrgyzstan (Zaitzev 1994). However, *L.montanosilvatica* is said to have unmarked wings, so *L.arcana* is considered a distinct species pending further revision of this genus.

*Leiabeckeri* is similar in colour to *L.arcana*; in examined specimens of *L.beckeri*, the dark-brown thoracic stripes are more sharply contrasted with the yellow sides and humeral area of the mesonotum than in *L.arcana*, and the pleura and abdomen are all dark brown. The marking over r-m may sometimes be faint in *L.beckeri*, but it differs in the preapical wing marking being situated closer to the tip of vein R_1_ than in the other Moroccan species and well before the tip of cell r_1_. The male terminalia of *L.beckeri* (Figs 11, 14, 17) are also similar to this group of the genus; the gonostylus is constructed similar to *L.bimaculata* and *L.arcana*, with the apical part intermediate in thickness between these species, but there is a slender tapered process ventral and external to the gonostylus (arrowed in Fig. 17), and the ventral lobe of the gonocoxites is broadly rounded and not apically produced as in these other species.

The females of these three species are similar in the structure of the ovipositor (Figs 18–20), but they evidently differ in the form in lateral view of the upper margin of sternite 8, which is more rounded in *L.bimaculata*, straighter and slightly emarginate in *L.arcana*, and with a more distinct emargination in *L.beckeri*.

##### Description.

**Figures 8, 9.**
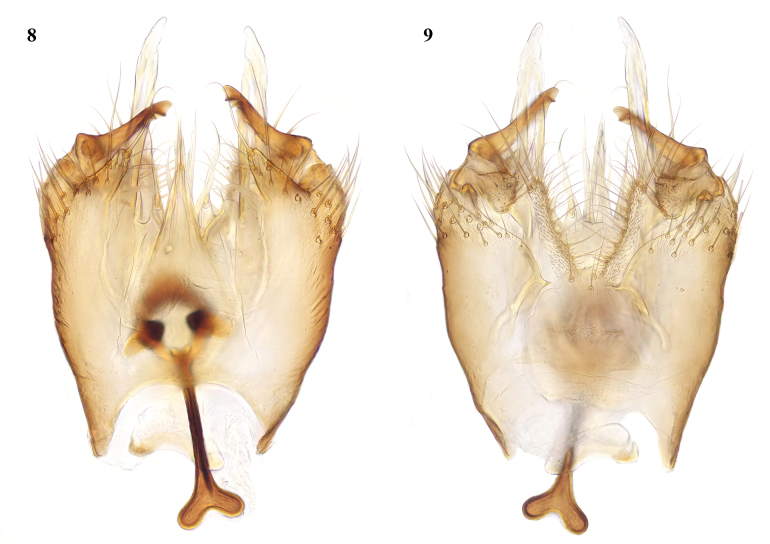
*Leiaarcana* sp. nov., male terminalia **8** ventral view **9** dorsal view.

**Male.** Wing length 4–4.5 mm. ***Coloration.*** Mainly shining black or dark brown with yellow markings. Head black; antenna with scape and pedicel yellow, flagellum dark brown. Mesonotum bearing three almost fused shining dark brown stripes (separated by narrow yellow dorsocentral stripes), leaving the humeral area and sides yellow; scutellum dark brown dorsally, sometimes more or less yellowish at sides; propleura brownish yellow; pleura otherwise and mediotergite all dark brown. Legs yellow except for narrow dark tips to coxae and trochanters and apical eighth of hind femur. Wing clear yellowish except for brown preapical patch that extends from fore margin (including tip of cell r_1_) over the median fork. Haltere yellowish white. Abdomen dark brown with hind margins of tergites 2–4 and fore margins (basal third) of tergites 3–5 narrowly yellow; sternites similarly coloured or with more yellow. Terminalia yellow. ***Head***. Antenna about 1.5× as long as head and thorax, with flagellomeres more than 2× as long as broad. ***Thorax.*** Mesonotum and scutellum with long yellow setae; laterotergite setose. ***Legs.*** Tibiae 2 and 3 with long yellow apical spurs, more than half as long as tarsomere 1; setulae on femora pale, on rest of legs dark; dark tibial setae, mostly longer than width of tibia: mid tibia with 2–3 d, 1 a-d, 3 a, and 2 a-v setae; hind tibia with 3–4 d and 3–4 a-d setae. ***Wing.*** Vein Sc ends in costa near to level of base of posterior fork, with crossvein sc-r at about its apical third. Vein R_1_ a third to half the length of crossvein r-m, which is longer than the stem of median fork. Median fork complete. Posterior fork arises before level of base of stem of median fork, its anterior branch (M_4_) narrowly interrupted at its extreme base. CuP stops short beyond level of base of posterior fork. A short dark streak at base of fork of axillary veins (as in *L.bimaculata*). ***Terminalia*** (Figs 8, 9, 12, 15). Gonostylus comprising a single lobe, curved medially and with a small preapical incision; gonocoxites with a setose ventral lobe basal to each gonostylus that is tapered to a bluntly rounded tip (arrowed in Fig. 15).

**Figures 10, 11.**
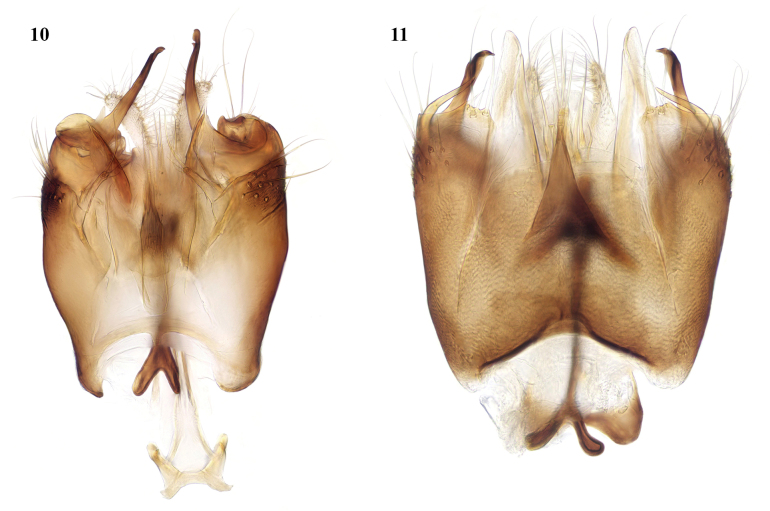
*Leia* species, male terminalia, ventral view **10***L.bimaculata* (Meigen, 1804) **11***L.beckeri* Landrock, 1940.

**Figures 12–14.**
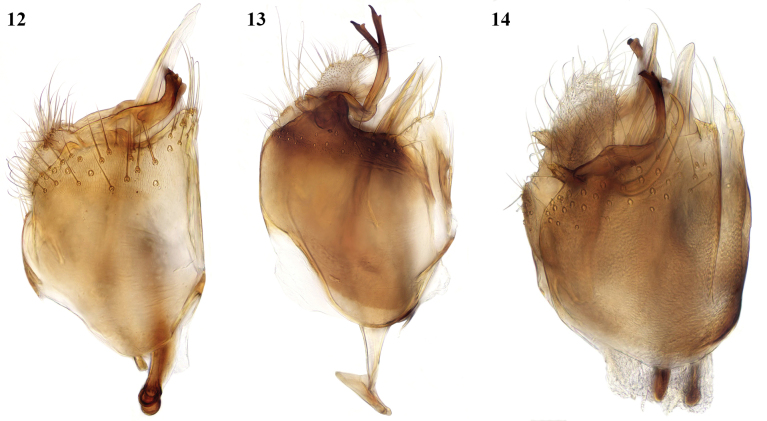
*Leia* species, male terminalia, lateral view **12***L.arcana* sp. nov. **13***L.bimaculata* (Meigen) **14***L.beckeri* Landrock.

**Figures 15–17.**
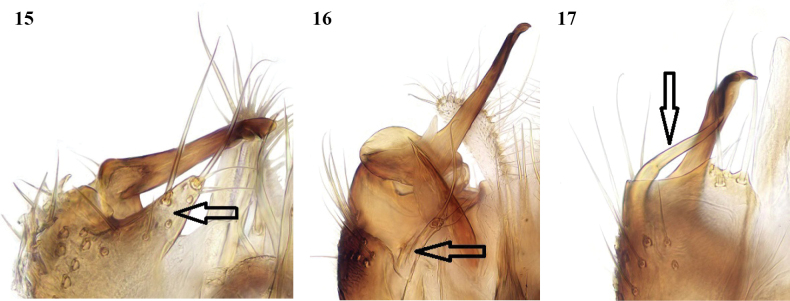
*Leia* species, male terminalia, ventral view of medial lobe of gonocoxite (arrowed in 15 and 16) and gonostylus **15***L.arcana* sp. nov. **16***L.bimaculata* (Meigen) **17***L.beckeri* Landrock (additional process from base of gonostylus arrowed, absent in other two species; medial lobe of gonocoxites broadly rounded).

**Figures 18–20.**
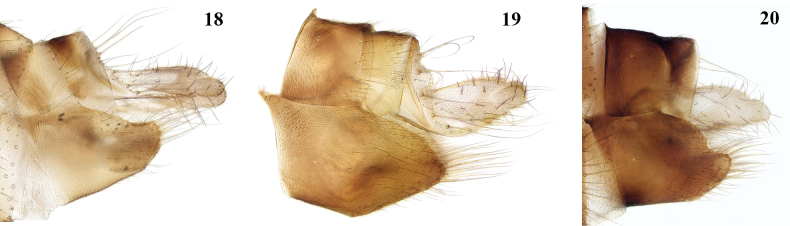
*Leia* species females, lateral view of ovipositor **18***L.arcana***19***L.bimaculata***20***L.beckeri*.

**Female. *Coloration.*** Similar to male, with scape and pedicel yellow, flagellum dark. Abdomen with segments 2–6 yellow on apical quarter; ovipositor with cerci narrow, brownish. ***Head.*** Antenna distinctly shorter than in male, less than length of thorax. ***Legs.*** Mid tibia with 3 d, 1 a-d, 3 v, and 3 p-v setae; hind tibia with 4 d, 3 a-d setae. ***Ovipositor*** (Fig. 18). Sternite 8 with dorsal margin in lateral view straight and cerci narrow.

##### Etymology.

From Latin *arcanus*, meaning secret as the separation of this species was previously hidden.

##### Comments.

The male from Aïn El Ma Bared and the female from Azrou were listed under *L.bimaculata* by Banamar et al. (2020). The female (Fig. 18) is considered conspecific with *L.arcana* on basis of its coloration.

**Genus *Megophthalmidia* Dziedzicki, 1889**

This genus was recognised to have a greater diversity in southern Europe than previously appreciated when six species, five of them newly described, were recorded from Greece by Chandler et al. (2006). Two of these species were recorded from Sardinia by Chandler (2009) and one of them, *M.illyrica* Chandler, Bechev & Caspers, 2006 is newly recorded here from Gibraltar (Governor’s lookout, Upper Rock, 1♂, 28/II/2010, coll. K. Bensusan and R. Gwillem). As there is also a species of this genus, *M.decora* (Santos Abreu, 1920), in the Canary Islands and Madeira the occurrence of the genus in Morocco was expected.

#### ﻿ Megophthalmidia amsemlili

Taxon classificationAnimaliaDipteraMycetophilidae

Chandler, Belqat & Banamar sp. nov.

EB76F5A3-DDAA-51CD-9A6B-BEB96125AF9D

https://zoobank.org/C4EF93E3-83ED-4461-86B7-CD09F33CD522

##### Type material.

***Holotype*.** Morocco – **Rif Region** • ♂ (mounted in DMHF, terminalia on slide); Marabout Abou Bnar; 18/V/2014; B. Belqat and O. Driauach leg; collected using sweep net; NHMUK. ***Paratypes.*** Morocco – **Rif Region** • 1♂; Daya Amsemlil; 26/III/2016; B. Belqat and O. Driauach leg; collected using sweep net; UAE-FST R16/2407 • 1♂; Forêt Malâab Tizimezzan; 12/V/2022; M.A. El Mouden leg; collected using sweep net; UAE-FST R22/2408 • 1♂; Pont Imezzane; 12/V/2022; M.A. El Mouden leg; collected using sweep net; UAE-FST R22/2409 • 1♂; Route vers Abou Bnar; 12/V/2022; M.A. El Mouden leg; collected using sweep net; UAE-FST R22/2410 • 1♂; Forêt Azilane; 13/V/2022; M.A. El Mouden leg; collected using sweep net; UAE-FST R22/2411 • 1♂; Daya Mtahen; 5/VI/2022; B. Belqat, M.A. El Mouden and O. Driauach leg; collected using sweep net; UAE-FST R22/2412. – **Middle Atlas Region** • 1♂; Oued Taourirt; 20/V/2022; M.A. El Mouden leg; collected using sweep net; UAE-FST MA22/2401. ***Other material.*** Morocco – **Rif Region** • 1♀; Daya Amsemlil; 23/IV/2016; B. Belqat and O. Driauach leg; collected using sweep net; UAE-FST R16/2413. – **Atlantic Plain Region** • 1♀; Sidi Yahya El Gharb; 25/IV/2015; B. Belqat and O. Driauach leg; collected using sweep net; UAE-FST AP15/2401.

Found in environments of protected areas (National Park of Talassemtane and Bouhachem Natural Park Project), around aquatic systems (rivers and ponds) but also in developed as well as inhabited areas.

##### Diagnosis.

This species is similar in coloration to *M.illyrica*, and the male tergite 9 is also similar in form to that species. The structure of the terminalia is otherwise quite distinct with the gonocoxites more deeply excavated ventrally and the gonostylus differing in form, broader basally and more angular apically. Specimens examined vary in the extent to which the gonostylus is extended in situ, giving a differing appearance which might suggest that more than one species is involved but their structural details are in common as described below. To take this apparent variation in form into account three specimens have contributed to the figures as indicated.

##### Description.

**Figures 21, 22.**
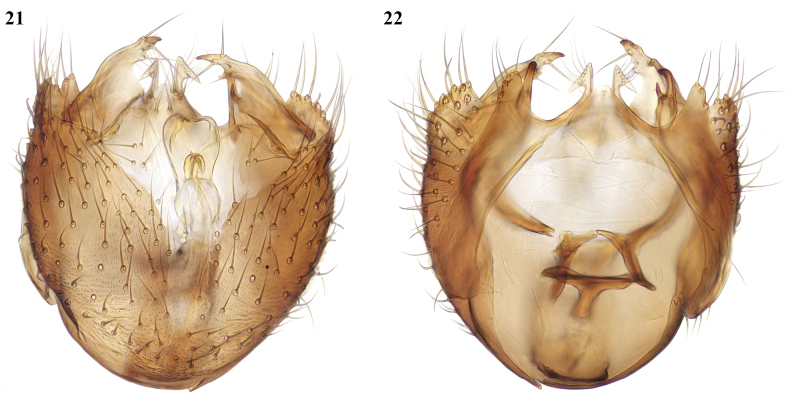
*Megophthalmidiaamsemlili* sp. nov., male terminalia, paratype from Pont Imezzane **21** ventral view **22** dorsal view.

**Figures 23–26.**
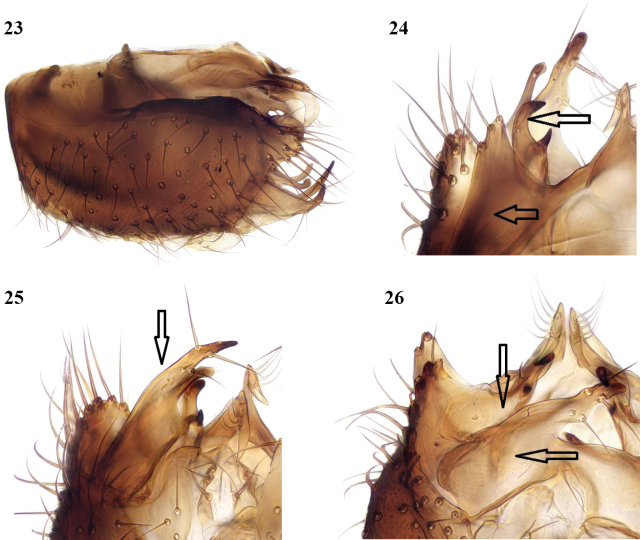
*Megophthalmidiaamsemlili* sp. nov., male terminalia **23** lateral view **24** dorsal view with tergite 9 (lower arrow) and gonocoxal margin (upper arrow) **25** ventral view, gonostylus (arrowed) extended posteriorly **26** ventral view, gonostylus (lower arrow) deflected internally, gonocoxal margin upper arrow **23–25** paratype from Oued Taourirt **26** holotype.

**Male.** Wing about 2 mm. ***Coloration.*** Body nearly all black, with yellowish apical margins to tergites 2–4, interrupted dorsally, and there may also be very narrow yellow basal margins to tergites 3–5; sternites 2–4 all yellow. Antenna black. Palpus black at base, otherwise yellow. Legs with mid and hind coxae brownish externally, otherwise all yellow. Wing clear yellowish. Terminalia dark coloured. ***Head.*** Antenna a little longer than head and thorax together, with flagellomeres at least as long as broad: flagellomeres 1 and 2 quadrate, other flagellomeres a little longer than broad. ***Legs.*** [Only one fore leg, femur, and part of tibia of one mid leg, and both hind legs are present in the holotype; paratypes are all missing one or more legs]. Mid tibia and hind tibia with rows of anterior and dorsal setae, all shorter than tibial width, the dorsal setae on hind tibia denser and occupying most of its length. Tibial spurs 1: 2: 2, yellow, the longer spurs on each about two-thirds length of first tarsomere. ***Wing.*** Radial veins and crossvein r-m with setulae, fork veins and their stems bare. Vein R_1_ a little longer than r-m, median stem about twice length of r-m. Base of posterior fork level with or just beyond base of stem of median fork, its branches widely divergent from base. Costa extends about 0.6 distance from R_4+5_ to M_1_. ***Terminalia*** (Figs 21–26). Small. Tergite 9 comprising apically pointed lateral lobes, connected by a narrow bridge to a prominent bilobed median process bearing cerci; each cercus with a ventrally directed tapered lobe with a row of long setae; gonocoxites with broad and deep medial excavation ventrally, dorsally produced medially on each side into a bifid process (arrowed in Figs 24, 26) with each lobe with a short terminal spine. Gonostylus (arrowed in Figs 25, 26) broad and bare basally, sharply narrowed to an angular apical part bearing some long setae and a short terminal tooth-like spine.

**Female.** Those listed under other material, with coloration as in male, and with flagellomeres similar to male, are considered likely to be conspecific.

##### Etymology.

Named for the locality Daya Amsemlil, where both *Megophthalmidia* species recorded here and the new species of *Mycomya* described below were collected.

##### Comments.

This is evidently a widespread species in Morocco.

#### ﻿ Megophthalmidia ionica

Taxon classificationAnimaliaDipteraMycetophilidae

Chandler, Bechev & Caspers, 2006

27D624BB-1DFF-5A3E-926C-6F34D9BCA1ED

##### New records.

Morocco – **Rif Region** • 1♂; Daya Amsemlil; 28/II/2015; B. Belqat and O. Driauach leg; collected using sweep net; UAE-FST R15/2443 • 1♂; Forêt Bouhachem st. 1; 5/V/2022; B. Belqat, M.A. El Mouden and O. Driauach leg; collected using sweep net; UAE-FST R22/2444 • 1♂; Daya Mtahen; 5/VI/2022; B. Belqat, M.A. El Mouden and O. Driauach leg; collected using sweep net; UAE-FST R22/2445.

##### Comments.

**Figures 27–29.**
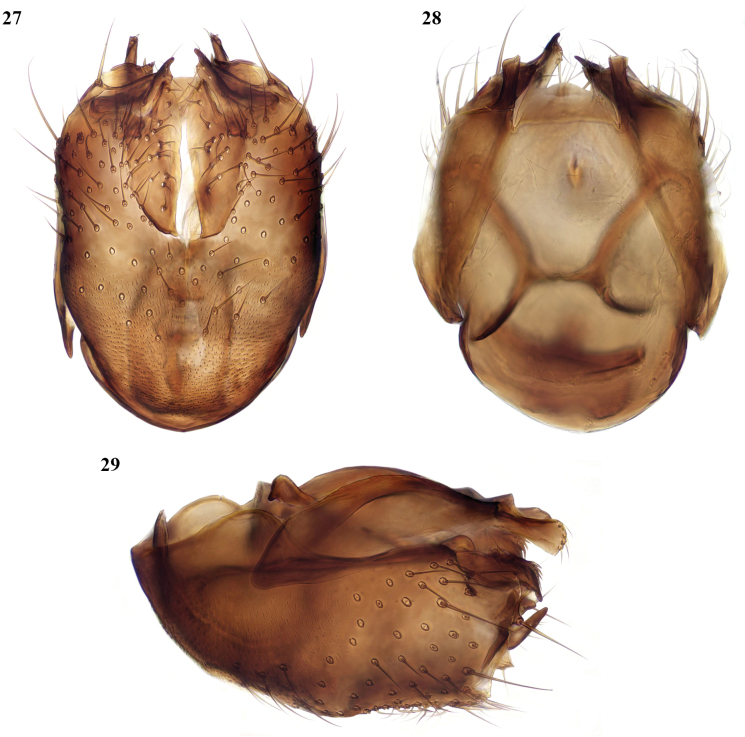
*Megophthalmidiaionica* Chandler, Bechev & Caspers, male terminalia **27** ventral view **28** dorsal view **29** lateral view.

This species was described from Greece (Chandler et al. 2006), later recorded from Sardinia (Chandler 2009), and has most recently been identified from Corsica. It is similar in coloration and most structural characters to *M.amsemlili* and *M.illyrica*. The terminalia (Figs 27–29) are, however, quite distinct in structure. The antenna is shorter than in *M.amsemlili* and *M.illyrica*; the flagellomeres, except the terminal one, are distinctly shorter than broad, and this character also enables females to be separated from those of *M.amsemlili* and *M.illyrica*. New to North Africa.

### ﻿Subfamily Gnoristinae Edwards,1925

**Genus *Docosia* Winnertz, 1864**

Species in this genus mostly have a uniform appearance of black body, mainly yellow legs and unmarked wings, specific characters being in small details of the structure of the male terminalia. Ševčík et al. (2020) noted that 57 species were now known from the Palaearctic region; of these about 20 have been recorded from around the Mediterranean, many of them little known apart from their original description. When the previous account of Moroccan Mycetophilidae (Banamar et al. 2020) was prepared, the material of *Docosia* had not yet been fully investigated, only the distinctive species *D.gilvipes* (Haliday in Walker, 1856) then being recorded. Although a larger number might be anticipated, so far five further species have been recognised in the available material, two of which are recorded here; the others are apparently previously undescribed and will be treated elsewhere. Females taken with males might be assumed to be conspecific but cannot be recognised for most species.

#### ﻿ Docosia melita

Taxon classificationAnimaliaDipteraMycetophilidae

Chandler & Gatt, 2000

08C38D77-2271-503C-A5EF-F2DF31702932

##### New records.

Morocco – **Rif Region** • 1♂; Oued Ferda; 13/II/2013; B. Belqat and O. Driauach leg; collected using sweep net; UAE-FST R13/2434 • 1♂; Oued Majjou avant source; 9/IV/2013; B. Belqat and O. Driauach leg; collected using sweep net; UAE-FST R13/2435 • 6♂♂; Maison forestière, Talassemtane; 17/VI/2014; B. Belqat and O. Driauach leg; collected using sweep net; UAE-FST R14/2436 • 2♂♂; Akchour; 16/IV/2016; B. Belqat and O. Driauach leg; collected using sweep net; UAE-FST R16/2437 • 1♂; Oued Majjou; 5/II/2019; B. Belqat and O. Driauach leg; collected using sweep net; UAE-FST R19/2438 • 2♂♂; Maison forestière, Talassemtane; 13/II/2021; B. Belqat and O. Driauach leg; collected using sweep net; UAE-FST R21/2439 • 3♂♂; Oued Majjou; 6/II/2022; B. Belqat and O. Driauach leg; collected using sweep net; UAE-FST R22/2440.

##### Comments.

**Figures 30, 31.**
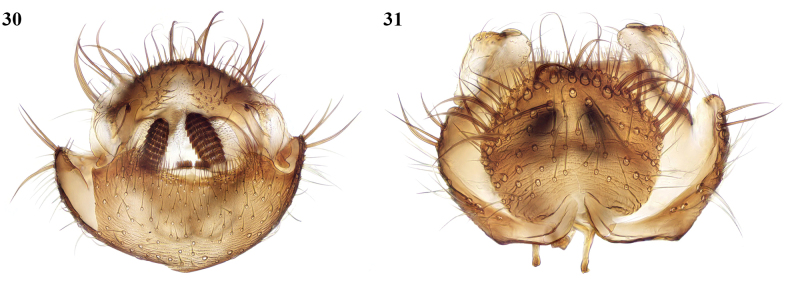
*Docosiamelita* Chandler & Gatt, male terminalia **30** ventral view **31** dorsal view.

This species was described from Malta and has been recorded from Greece (Chandler et al. 2006) and Sardinia (Chandler 2009). It can also be newly recorded from Gibraltar (Mediterranean steps, maquis, 1♂, 23/III/2010; Camp Bay, ruderal vegetation, 1♂, 21/III/2010, both coll. M.J. Ebejer). New to North Africa. The male terminalia of a Moroccan specimen are shown here (Figs 30, 31); the female cannot be distinguished from allied species.

#### ﻿ Docosia flavicoxa

Taxon classificationAnimaliaDipteraMycetophilidae

Strobl, 1900

D4FF1013-38FB-54A9-8C7B-0C8E350E7D02

##### New records.

Morocco – **Rif Region** • 1♂; Oued Ferda; 13/II/2013; B. Belqat and O. Driauach leg; collected using sweep net; UAE-FST R13/2428 • 2♀; Oued Majjou avant source; 9/IV/2013; B. Belqat and O. Driauach leg; collected using sweep net; UAE-FST R13/2429 • 1♂; Daya avant Taïda; 20/IV/2018; B. Belqat and O. Driauach leg; collected using sweep net; UAE-FST R18/2430 • 1♂; Oued Majjou; 5/II/2019; B. Belqat and O. Driauach leg; collected using sweep net; UAE-FST R19/2431 • 2♀♀; Oued Majjou; 6/II/2022; B. Belqat and O. Driauach leg; collected using sweep net; UAE-FST R22/2432 • 1♂; Oued Majjou; 20/II/2022; B. Belqat and O. Driauach leg; collected using sweep net; UAE-FST R22/2433.

##### Comments.

**Figures 32, 33.**
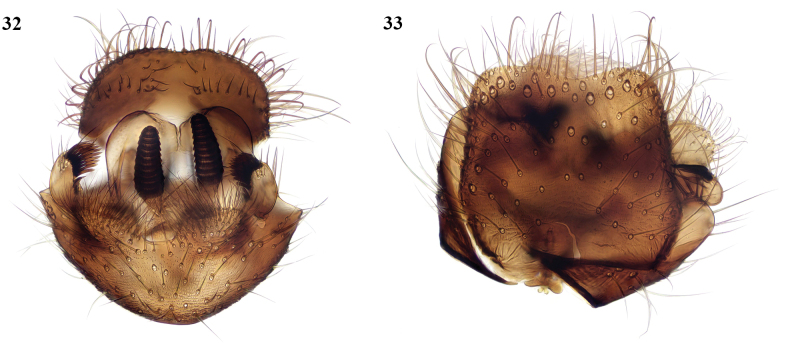
*Docosiaflavicoxa* Strobl, male terminalia **32** posteroventral view **33** dorsal view.

This is a widespread Palaearctic species, distinguished by its entirely yellow legs from the other Moroccan species which have the bases of the mid and hind coxae more or less darkened, and also by its setose laterotergite which is bare in the other Moroccan species examined. It is also newly recorded from Gibraltar (Upper Rock, meadow in woodland, 1♂, 1♀, 21/III/2010, coll. K. Bensusan). New to North Africa. The terminalia of a Moroccan specimen are shown here (Figs 32, 33).

### ﻿Subfamily Mycomyinae Edwards, 1925

**Genus *Mycomya* Rondani, 1856**

#### ﻿ Mycomya mira

Taxon classificationAnimaliaDipteraMycetophilidae

Chandler, Belqat & Banamar sp. nov.

0A0611C0-15EF-55E2-9BE9-2159142C99B6

https://zoobank.org/BBE4B953-B1BF-4B5D-9292-48843474873B

##### Type material.

***Holotype*.** Morocco – **Rif Region** • ♂ (mounted in DMHF); Forêt Adayourha; 1/II/2020; B. Belqat and O. Driauach leg; collected using sweep net; NHMUK. ***Paratypes.*** Morocco – **Rif Region** • 2♂♂; Forêt Bab Hajara; 14/VII/2013; B. Belqat and O. Driauach leg; collected using sweep net; UAE-FST R13/2414 • 1♂; Aïn Sidi Brahim Ben Arrif; 25/IV/2014; B. Belqat and O. Driauach leg; collected using sweep net; UAE-FST R14/2415 • 5♂♂, 2♀♀; Forêt Bab Hajara; 28/II/2015; B. Belqat and O. Driauach leg; collected using sweep net; UAE-FST R15/2416 • 32♂♂; Daya Amsemlil; 1/I/2020; B. Belqat and O. Driauach leg; collected using sweep net; UAE-FST R20/2417 • 19♂♂, 4♀♀; Forêt Adayourha; 1/II/2020; B. Belqat and O. Driauach leg; collected using sweep net; UAE-FST R20/2418 • 5♀♀; Maison forestière Bouhachem; 1/II/2020; B. Belqat and O. Driauach leg; collected using sweep net; UAE-FST R20/2419 • 7♂♂, 1♀; Forêt Sed Nakhla; 10/II/2020; B. Belqat and O. Driauach leg; collected using sweep net; UAE-FST R20/2420 • 1♂; Forêt Azilane; 13/II/2020; B. Belqat and O. Driauach leg; collected using sweep net; UAE-FST R20/2421 • 1♂; Forêt Bni Leit; 10/I/2021; B. Belqat and O. Driauach leg; collected using sweep net; UAE-FST R21/2422 • 1♂; Douar Bni Leit; 10–16/I/2021; B. Belqat and O. Driauach leg; reared; UAE-FST R21/2423.

This species inhabits the diverse landscape of the Bouhachem Natural Parc Project, particularly including wetlands, which present a typology from sphagnum peat bogs to temporary ponds to springs, spring streams, and headwaters of three river systems, as well as in forests.

##### Diagnosis.

This species belongs to the subgenus Mycomya sensu stricto and differs from other species in the following combination of male characters: legs simple except for short mid-coxal spur; tergite 9 with neither a medial process nor lateral appendages, medially emarginate and with short internal spinose setae subapically. It runs to couplet 75 in the key by Väisänen (1984), where it is between the two options in that the base of the posterior fork is usually level with the base of the stem of the median fork. It fits the first option in the structure of tergite 9 and in couplet 76 it agrees with the western Nearctic *M.fuscipalpis* van Duzee, 1928 in the form of the gonostylus. Thus, it could be assigned to the species group of which *M.fuscipalpis* was the only member. The most obvious difference from *M.fuscipalpis* is that there are separate submedian appendages of the gonocoxites, while in *M.fuscipalpis* these are fused medially.

##### Description.

**Figures 34, 35.**
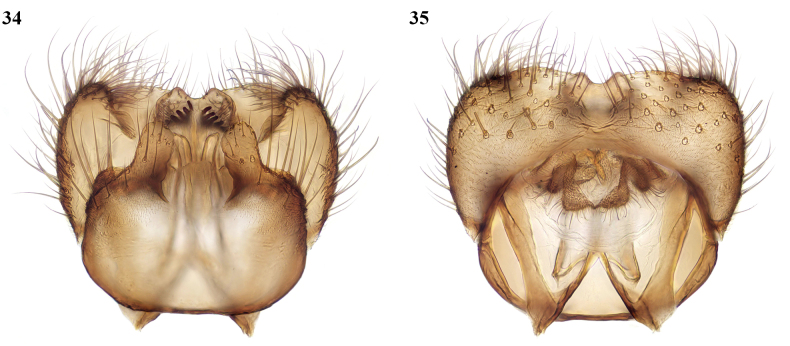
*Mycomyamira* sp. nov., male terminalia **34** ventral view **35** dorsal view.

**Male.** Wing length 4–4.5 mm. ***Coloration.*** Body entirely dark greyish brown. Head and antennae uniformly dark; palpi yellowish. Coxae brown, legs otherwise entirely yellow. Terminalia dark grey. ***Head.*** Antenna slender, about 3 mm long, longer than abdomen, with flagellomeres about 6× as long as broad. **Legs.** Long and slender. Fore coxa unmodified; mid coxa with anteriorly directed slender spur, straight for most of its length, then slightly curved apically, relatively short, about half length of coxa. Fore tarsomere 1 a little shorter than its tibia. Vein Sc ending in R at middle of radial cell, often with anterior spur, more or less extended to costa (may vary between the wings of a specimen). Base of posterior fork at or just beyond level of base of stem of median fork. ***Terminalia*** (Figs 34, 35). Short. Tergite 9 with a median emargination between broad rounded setose lobes, with a pair of internal submedian lobes each bearing a slender curved spine laterally and a row of 5–7 short blunt spines (cones of Väisänen 1984) apically. Gonocoxites with short broad submedian appendages that are bluntly rounded apically. Gonostylus reflexed within gonocoxites, thick and angular basally and sharply narrowed to a slender curved and pointed apical part which has a small blunt tooth at its base.

**Figures 36.**
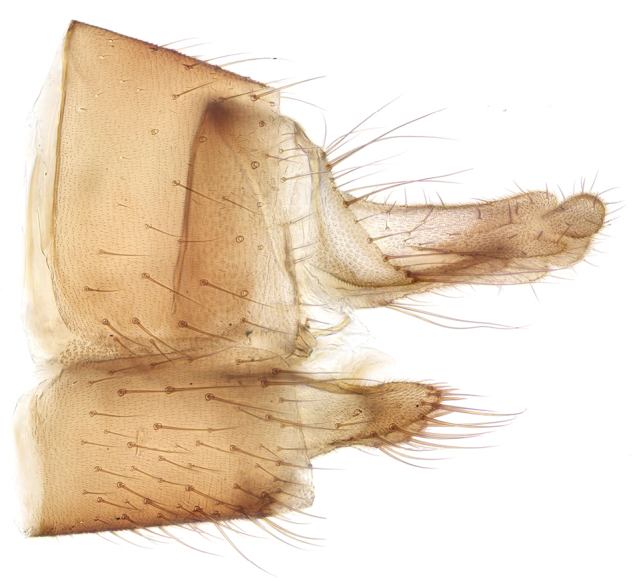
*Mycomyamira* sp. nov., female, lateral view of ovipositor.

**Female.** Wing length range as in male. ***Coloration*.** As in male; ovipositor yellowish. ***Head.*** Antenna relatively shorter than in male, about 1.5× head and thorax together; flagellomeres about 4× as long as broad. ***Legs.*** Simple, without mid-coxal spur. ***Ovipositor*** (Fig. 36). Sternite 8 with a pair of tapered apically rounded setose lobes. Cercus with elongate basal segment and small rounded apical segment bearing short setae.

##### Etymology.

From Latin *mirus*, to note the astonishing discovery of this species.

##### Comments.

This is a very distinct species, which is evidently frequent and widespread in Morocco.

#### ﻿ Mycomya prominens

Taxon classificationAnimaliaDipteraMycetophilidae

(Lundström, 1913)

3EA81CA8-1B7B-533D-8E25-C2F897CDC81B

##### New records.

Morocco – **Rif Region** • 1♂; Daya Mtahen; 23/III/2021; B. Belqat and O. Driauach leg; collected using sweep net; UAE-FST R21/2448.

##### Comments.

This is a common and widespread European species, with previous records in the Mediterranean region from Israel and Greece (Chandler 1994; Chandler et al. 2006). It has also been recorded from Madeira (Chandler and Ribeiro 1995). New to North Africa.

### ﻿Subfamily Sciophilinae Rondani, 1840

**Genus *Monoclona* Mik, 1886**

#### ﻿ Monoclona rufilatera

Taxon classificationAnimaliaDipteraMycetophilidae

(Walker, 1837)

D449A7F7-E2F4-547B-BAA8-CFF364282A8B

##### New records.

Morocco – **Rif Region** • 1♂; Forêt Aïn Lahcen; 15/I/2020; B. Belqat and O. Driauach leg; collected using sweep net; UAE-FST R20/2446 • 1♂; Khandek Melouka; 10/IV/2021; B. Belqat and O. Driauach leg; collected using sweep net; UAE-FST R21/24467.

##### Comments.

This is a Holarctic species, which is widespread in Europe. New to North Africa.

**Genus *Phthinia* Winnertz, 1864**

This is a small genus, but with a diversity of structure of the male terminalia. Zaitzev (1994) included 10 Palaearctic species, of which four occur in Europe, one (*P.hyrcanica* Zaitzev, 1984) occurs in Azerbaijan, and the rest live in the Eastern Palaearctic. Three more European species have since been described (Plassmann 1984, 1990; Zaitzev 2001). A male and a female, collected at the same locality in Morocco, have been examined; both specimens are in poor condition, but the structure of the male terminalia is distinct from any previously known species of the genus.

#### ﻿ Phthinia snibbypinsae

Taxon classificationAnimaliaDipteraMycetophilidae

Chandler, Belqat & Driauach sp. nov.

11CCF3E4-B56A-530F-BDCA-31C2FA0866DC

https://zoobank.org/C90346E3-F2FF-40A7-8F37-092DD7421877

##### Type material.

***Holotype*.** Morocco – **Rif Region** • ♂ (mounted in DMHF, terminalia on slide); Khandek Melouka; 10/IV/2021; B. Belqat and O. Driauach leg; collected using sweep net; NHMUK. ***Paratype.*** ♀; same data as for holotype; UAE-FST R21/2424.

The type locality is in the environment (forest and cultivated fields) of Aïn Lahcen, a rural commune whose name (Aïn) is taken from a spring that flows through it.

##### Diagnosis.

This is a slender bodied species with small male terminalia, similar in this respect to *P.winnertzi* Mik, 1869 and allied species. Among Palaearctic species, it most closely resembles *P.hyrcanica* in the apically bilobed gonostylus situated within the broadly rounded gonocoxites. It differs from that species in the lobes of the gonostylus being short and blunt and in the dense short setae on the margins of the gonocoxites.

##### Description.

**Figures 37, 38.**
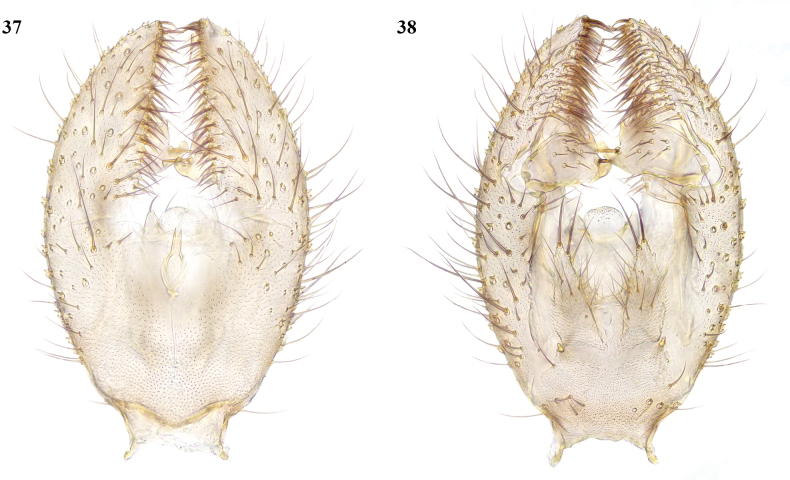
*Phthiniasnibbypinsae* sp. nov., male terminalia **37** ventral view **38** dorsal view.

**Male.** Body 7 mm, of which about 6 mm is length of abdomen. ***Coloration.*** Head brown. Antenna with short basal segments and base of first flagellomere yellow, remainder brown. Palpi yellow. Thorax yellowish brown, darker brown on disc of mesonotum and scutellum. Legs yellow. Wings clear grey, presumed to be unmarked as in female. Haltere brown. Abdomen entirely dark brown. Terminalia yellow. ***Head.*** Both antennae incomplete (11 and 5 flagellomeres present). ***Legs.*** Missing apart from one fore femur and one hind leg. ***Wing.*** Both wings are represented only by short stubs. ***Abdomen.*** Long, slender. ***Terminalia*** (Figs 37, 38). Small. Gonocoxites rounded laterally, with deep medial excavation bordered by short, dense setae; gonostylus enclosed by gonocoxites, short and with two short, blunt apical lobes.

**Female.** Body 6 mm, of which around 5 mm is length of abdomen. ***Coloration***. As in male; wings clear greyish. Ovipositor brownish yellow. ***Legs.*** Fore legs missing but mid and hind legs complete, long, slender, about 9 mm long. ***Wing.*** Both wings are represented only by stubs, but more of the right wing is present, including the bases of fork veins. Vein Sc ends in costa before level of base of Rs. Crossvein r-m about 3× as long as stem of median fork. Base of posterior fork beyond that of median fork, with posterior branch (vein CuA) downturned; false vein also downturned, parallel with it. Vein CuP stops short before level of base of posterior fork. ***Abdomen.*** Slender, relatively shorter than in male. Ovipositor short and small, with cerci short ovoid and covered with short setae.

##### Etymology.

The name commemorates Snibby Pins, erstwhile companion of Benjamin Bottom, after whom the Sardinian gnat *Sciophilabenjaminbottomi* Chandler, 2009 was named.

##### Comments.

This is the first record of this genus from North Africa, and this species is evidently rare. *Phthinia* species are usually found around rotten wood, and they develop in encrusting fungi.

## ﻿Discussion

The new findings presented in this study increase the number of Mycetophilidae of Morocco to 76 species, so enriching the biodiversity of the Mycetophilidae fauna of the whole North Africa region. The fauna of other parts of North Africa is poorly known, with only 23 species of Mycetophilidae presently recorded from Algeria and 27 species from Tunisia, with a combined total including unpublished records of 45 species, of which 26 are in common with Morocco; this comparison will be discussed further elsewhere. The five newly described species allow us to consider for the first time endemic mycetophilids in Morocco, of which three are specifically endemic to the occidental Rif region. More fieldwork in this region and elsewhere in Morocco will probably find more new species.

## Supplementary Material

XML Treatment for Exechia repandoides

XML Treatment for Exechiopsis corona

XML Treatment for Rymosia ebejeri

XML Treatment for Leia arcana

XML Treatment for Megophthalmidia amsemlili

XML Treatment for Megophthalmidia ionica

XML Treatment for Docosia melita

XML Treatment for Docosia flavicoxa

XML Treatment for Mycomya mira

XML Treatment for Mycomya prominens

XML Treatment for Monoclona rufilatera

XML Treatment for Phthinia snibbypinsae

## ﻿Additional information

### Author ORCIDs

Mohamed Amin El Mouden https://orcid.org/0009-0002-6085-924X

Peter J. Chandler https://orcid.org/0009-0000-4789-3596

Ouafaa Driauach https://orcid.org/0000-0002-9748-2450

Ouarda Banamar https://orcid.org/0000-0002-7838-1080

Imane Saidoun https://orcid.org/0009-0005-2809-5384

Abdellatif Akarid https://orcid.org/0000-0003-3823-1229

Khalid Aattouch https://orcid.org/0009-0001-0680-4822

Boutaïna Belqat https://orcid.org/0000-0003-2857-7699

### Data availability

All of the data that support the findings of this study are available in the main text.
